# Human Poisoning due to Delphinium species in the Himalayan Region of Nepal: A Case Report

**DOI:** 10.31729/jnma.5285

**Published:** 2020-12-31

**Authors:** Santosh Adhikari, Abhishek Bhandari

**Affiliations:** 1 Manang District Hospital, Manang, Nepal; 2 Sindhuli Hospital, Sindhuli, Nepal

**Keywords:** *alkaloids*, *Delphinium*, *neuromuscular blockade*, *poisoning*

## Abstract

The *Delphinium* species herb, common name ‘Nirmasi’ in Nepal, is one of the community level flower herbs used as medicinal ingredients in various clinical problems in Manang District and other Himalayan parts of Nepal. Roots of the plants from the genus Delphinium have been used for a long time for headache, epilepsy, mania, paralysis, rheumatism, toothache, and various types of pain. However, many species of Delphinium are poisonous and look quite similar in morphology to the beneficial ones. As a result, accidental poisoning is common. Poisoning due to these plants results in symptoms due to gastric irritation, competitive neuromuscular blockade, and cardiotoxicity caused by various alkaloids present in them. We report here a case of poisoning due to Delphinium species ingestion presenting as hypotension and bradycardia managed successfully with symptomatic treatment.

## INTRODUCTION

*Delpheniumbrunonianum,* D. himalayai, and D.stapeliosumare some of the species found in Nepal, India, and the Himalaya Mountains.^1^ Their roots have been used for the treatment of fever, headache, stomachache, jaundice, and skin rashes.^2^ However, some species are toxic and the toxicity of the genus Delphiniumis variable depending on growth stages and concentration of a toxic substance.^3^ Poisoning usually occurs in grazing cattle and is rare in humans. So, mistaking the medicinal species, a poisonous species may be ingested leading to poisoning in humans. This report details a case of Delphinium species poisoning presented with bradycardia and hypotension.

## CASE REPORT

A 32-year male without any past comorbid illness, presented in the emergency unit of Manang District Hospital, Chame, Manang, Nepal with a history of ingestion of herbal plant ‘Nirmasi’ (Delphinium) followed by multiple episodes of vomiting containing ingested food particles, generalized tingling, and burning sensation, restlessness, and agitation after three hours of ingestion. The patient had taken the plant product as a normal tonic intake. On presentation to an emergency, the patient was anxious but well oriented to time, place, and person, and was vomiting actively. The patient had a blood pressure of 80/60 mm of Hg, a heart rate of 53 beats/minute, an axillary temperature of 970 Fahrenheit, and a respiratory rate of 22 breaths per minute, and capillary oxygen saturation of 96% in room air. Systemic examination, including the cardiovascular and respiratory system, was normal.

**Figure 1:**
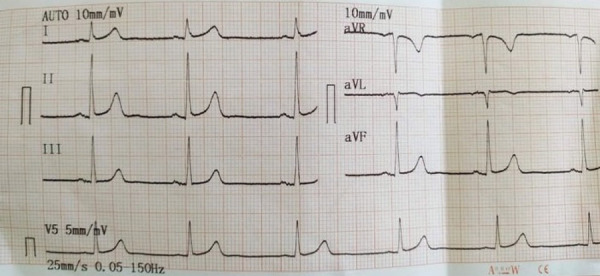

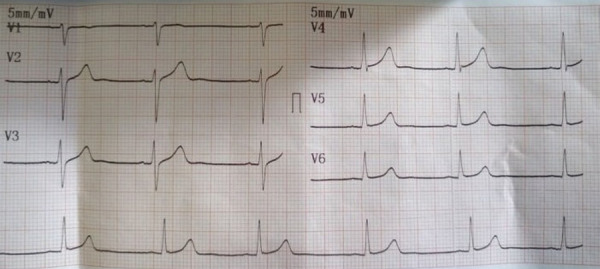
Electrocardiogram showing sinus bradycardia.

Immediate twelve lead ECG showed sinus bradycardia with a rate of 54 beats per minute (Figure 1).

All the investigations including hemoglobin, total leukocyte count, differential leukocyte count, serum sodium, potassium, urea, and creatinine, and serum level of liver enzymes were within the normal range (Table 1).

**Table 1:** Value of lab parameters.

Lab Parameters (units)	Value at admission
Total Leucocyte Count (cells/mm3)	7100
Differential Leucocyte count (% of Total Leucocyte count)	Neutrophil 65%
	Lymphocyte 35%
Hemogobin (g/dl)	13.6
Serum urea (mg/dl)	20
Serum Creatinine (mg/dl)	0.8
Serum Sodium (mmol/L)	138
Serum Potassium (mmol/L)	4.1
Serum Total Bilirubin (mg/dl)	1.4

The patient was given bolus intravenous crystalloids at 20ml/kg within 30 minutes and was kept in Atropine infusion to maintain mean arterial pressure above 70 mmHg and heart rate above 50 beats per minute. The patient was kept nil per oral and received maintenance fluid at 80 ml/hour, injectable proton pump inhibitor, and antiemetic. After eight hours of treatment, atropine infusion was withheld and his heart rate normalized to 70 to 75 beats per minute and twelve-lead electrocardiography showed normal sinus rhythm which persisted throughout the hospital stay. The patient also made adequate urine output of 60 ml per hour over this time and continued to do so during a hospital stay. Symptoms of vomiting also subsided over this time. The patient was discharged after 48 hours of observation in the medical ward without complications.

## DISCUSSION

Extracts from various Delphinium species like Delpheniumbrunonianum, D. himalayai, and D. stapeliosum namely (β-amyrin, (β-sitosterol, etc. exhibited antibacterial properties against various bacteria like Sstaphylococcus aureus, Pseudomonas aureginous, Escherchia coli, etc.^2^ However, other extracts like methylsuccimidoanthronyllycoctonine (MSAL) and the lycoctonine group of alkaloids found in Delphinium peregrinum have the toxic effect of causing neuromuscular paralysis via competitive inhibition of post-synaptic neurotransmitter acetylcholine, specifically acting at the α1 nicotinic sites in the brain and muscle, causing respiratory depression and other curare-like symptoms.^4,5^ Other Delphinium alkaloids like methyllycaconitine (MLA), nudicauline, barbinine, deltalineetc also block neuromuscular transmission by acting as nicotinic receptor antagonist.^5,6^ Our patient presented with complaints of dizziness secondary to bradycardia and hypotension which could be the effect of such alkaloids competitively inhibiting the acetycholine at preganglionic sympathetic synapse blocking the sympathetic outflow to the heart.

There has been no specific antidote noted for Delphinium poisoning in humans. However, physostigmine has been shown to reverse the effects of the alkaloids present in Delphinium in cattle.^7^ In our patient, symptomatic emergency care with appropriate fluids and low dose atropine improved hypotension and bradycardia within a short period. So, the patient's history of consumption of this Delphinium plant parts is ultimate for diagnosis in Nepal. So far, proper studies have not been done in Nepal to identify the medical value and toxicity of different species of Delphinium. This sort of accidental poisoning warrants such studies and the creation of awareness among residents of areas where Delphinium is a common medicinal plant. Also, with timely intervention and observation patients presenting with poisoning due to these plants can be treated with easily available and cheap drugs with good results. Physicians working in areas where homeopathic medicines are commonly used must look into these types of patients with a high index of suspicion.
